# Concerted Actions by PIICP, CTXII, and TNF-α in Patients with Juvenile Idiopathic Arthritis

**DOI:** 10.3390/biom11050648

**Published:** 2021-04-28

**Authors:** Katarzyna Winsz-Szczotka, Kornelia Kuźnik-Trocha, Iwona Lachór-Motyka, Wojciech Lemski, Krystyna Olczyk

**Affiliations:** 1 Department of Clinical Chemistry and Laboratory Diagnostics, Faculty of Pharmaceutical Sciences in Sosnowiec, Medical University of Silesia, ul. Jedności 8, 41-200 Sosnowiec, Poland; kkuznik@sum.edu.pl (K.K.-T.); lemski@wp.pl (W.L.); olczyk@sum.edu.pl (K.O.); 2 Department of Rheumatology, The John Paul II Pediatric Center in Sosnowiec, ul. Gabrieli Zapolskiej 3, 41-218 Sosnowiec, Poland; iwlamo@tlen.pl

**Keywords:** juvenile idiopathic arthritis, cartilage turnover markers, procollagen II C-terminal propeptide, C-telopeptide of type II collagen, tumor necrosis factor-α

## Abstract

Joint destruction in juvenile idiopathic arthritis (JIA), initiated in the early, preclinical stage of the disease, is diagnosed on the basis of clinical evaluation and radiographic imaging. The determination of circulating cartilage-matrix turnover markers can facilitate the diagnosis and application of better and earlier treatment strategies for JIA. We have shown that 96 JIA patients have elevated levels of procollagen II C-terminal propeptide (PIICP), reflecting the extent of joint cartilage biosynthesis, and C-telopeptide of type II collagen (CTXII), a biomarker of the resorption of this tissue. Patients who did not respond to treatment had particularly high levels of these markers. JIA treatment resulted in the normalization of these markers in remissive patients, but not in those with active JIA. We showed correlations between examined variables and inflammatory process indicators, i.e., C-reactive protein (CRP), erythrocyte sedimentation rate (ESR), and tumor necrosis factor-α (TNF-α). The TNF-α of patients responding to treatment correlated with PIICP, especially in the patients before treatment (r = 0.898, *p* < 0.001). Significant changes in serum PIICP during JIA therapy suggest its potential diagnostic utility in the monitoring of disease activity and the possibility of its use in assessing treatment towards remission. Understanding changes in type II collagen metabolism over the course of the discussed arthritis may allow the implementation of both new diagnostic tools and new therapeutic strategies in children with JIA.

## 1. Introduction

The term juvenile idiopathic arthritis (JIA) defines a heterogeneous collection of autoimmune or autoinflammatory rheumatic diseases, with onset before the age of 16 years. There is no specific symptom or examination findings for JIA, and diagnosis is made by exclusion and differentiation [1,2,3]. JIA that is poorly treated or diagnosed too late may contribute to the disability of an afflicted child, due to disturbances in the structure and function of the osteoarticular system. In particular, these disorders are attributed to changes in the homeostasis of extracellular matrix (ECM) components, of which the cartilage is composed [4,5,6].

The cartilaginous extracellular matrix is a multicomponent, ordered, flexible network structure that fills the spaces between chondrocytes [7,8]. The cartilage ECM consists of collagen proteins, among which type II collagen (CII) predominates, while types IX, X, XI, VI, XII, and XIV are present in smaller amounts. Mentioned collagen fibrils provide cartilage with tensile strength and contribute to the physical properties of the mature matrix. Moreover, the cartilage matrix is formed by proteoglycan aggregates, including aggrecan, decorin, biglycan, fibromodulin, lumican, and proteoglycan-100. Additionally, within the structure of this matrix there are small amounts of non-collagen proteins, including fibronectin, tenascin, chondronectin, vitronectin, thrombospondin, and matrilins. In a physiological state, these above-mentioned ECM components co-create dynamic equilibrium, in which old molecules are being degraded and new compounds are being synthesized continuously [7,8,9,10]. This balance may be disturbed in JIA, as the activity of ECM-degrading factors in these patients is high, including matrix metalloproteinases (MMP), a disintegrin-like and metalloproteinase with thrombospondin motifs (ADAMTS), or reactive oxygen species (ROSs), which are not equalized by the activity of factors stimulating ECM synthesis, including insulin-like growth factor-1 (IGF-1), transforming growth factor-β (TGF-β), or platelet-derived growth factor-BB (PDGF-BB) [3,6,7,8,9,10]. The lack of this balance can lead to irreversible degradation in the cartilage collagen network, which seems to be a critical event involved in the pathophysiological progress of arthritis. Alterations in the CII metabolism occur prior to detectable radiographic changes of joints [11]. The early detection of these collagen changes would allow clinicians to initiate relevant therapies essential for the long-term outcome of JIA. During the remodeling of the collagen network, small protein fragments are released into the circulation [8,9]. CII synthesis markers include the procollagen II C-terminal propeptide (PIICP) or the procollagen IIA N-propeptide (PIIANP), while CII degradation markers include C-telopeptide of type II collagen (CTX-II), CTX-II neoepitope (EKGPDPLQ), CII neoepitope, helix II, or C2C [11,12,13]. These fragments may be utilized as specific and early diagnostic or prognostic serological markers, as they originate from the structure of cartilage, whose metabolic disorder is partially the consequence of disease. For example, CTX-II has been shown to be a highly relevant biomarker of cartilage degradation in adult patients with rheumatic diseases, enabling the prediction of disease progression, treatment efficacy, bone mineral density reduction, and synovitis [12]. Serum PIICP values indicate type II collagen formation in normal and inflamed cartilage. In turn, the synovial levels of PIICP are increased in osteoarthritis patients in comparison to healthy individuals and correlate with the degree of joint space narrowing [13]. Thus far, markers of CII changes in children with JIA have not been assessed.

Given the above-mentioned data, the aim of our study was to evaluate the serum concentration of both PIICP, which reflects the extent of joint cartilage biosynthesis, and CTXII, as a biomarker of resorption of this tissue, in patients with newly diagnosed JIA and in the same patients both after clinical improvement, observed following an inflammation modifying therapy, as well as in patients in whom the therapy prescribed did not result in remission. Additionally, due to the significant involvement of tumor necrosis factor-α (TNF-α) in initiating the inflammatory processes underlying the development of JIA [14], the purpose of the study was also to assess the relation of this factor to cartilage markers of collagen type II turnover. We have also evaluated interactions between the cartilage markers, TNF-α concentrations, and inflammatory indicators, i.e., C-reactive protein (CRP) and erythrocyte sedimentation rate (ESR).

## 2. Materials and Methods

### 2.1. Patients and Samples

The research was carried out on the serum obtained from 96 Polish, Caucasian children of both sexes, i.e., 77 girls and 19 boys, aged 5–12 years, with newly diagnosed JIA (serum was frozen at −80 °C and thawed immediately prior to testing). The medical records of each case were reviewed by a pediatric rheumatologist on the investigational team. All the patients were diagnosed and classified as oligoarthritis or polyarthritis (as per International League of Associations for Rheumatology criteria) [15]. Moderate disease activity was assessed in all patients (according to the Juvenile Arthritis Disease Activity Score). Other forms of JIA, as well as any other chronic and autoimmune diseases, were considered the exclusion criteria. Moreover, the accuracy of the diagnosis was confirmed by laboratory tests, namely indicators of the inflammatory response, i.e., CRP (immunonephelometric assay) and ESR (Westergren method), measuring the rheumatoid factor (latex enhanced immunoturbidimetric test), and by determining the titer of antinuclear antibodies (indirect immunofluorescence assay) (Table 1).

The treatment with stable doses of non-steroidal anti-inflammatory drugs (ibuprofen and naproxen were the most commonly used agents), oral glucocorticoids (at a maximum dose of 1 mg of a prednisone equivalent per kilogram per day, with gradual dose reduction), sulfasalazine (30 mg per square meter of body-surface area), and methotrexate (≤15 mg per square meter of body-surface area once a week), was prescribed. No other disease-modifying anti-rheumatic drugs or biologic agents were allowed. Untreated JIA patients were divided into two groups, according to their demonstrated sensitivity to treatment, i.e., 30 JIA patients responding to treatment (A), and 66 JIA patients not responding to treatment (B). The aim of this study, the biochemical assessments, were repeated in all patients, i.e., in 66 patients in whom the therapy modifying the course of the disease failed to improve the clinical condition or was poorly tolerated, on average, 4.41 ± 0.86 months after the beginning of the therapy (B’—active disease), as well as in 30 patients after the therapy for whom clinical improvement was observed, on average, 10.02 ± 0.61 months after the beginning of the therapy (A’—inactive disease). Clinically inactive disease was diagnosed in patients when: there was no active joint disease; no fever, erythema, serositis, splenomegaly, diffuse lymphadenopathy, as well as uveitis; ESR and CRP were normal; morning stiffness lasted less than 15 min; and the physician visual analogue scale (VAS) was of the lowest value on the scale used. The 66 treated patients who did not show clinical improvement were classified for another therapy.

The reference material comprised of blood samples collected from 45 healthy children (34 girls, 11 boys), of ages matching respectively the JIA patients, i.e., before puberty, assessed according to the Tanner scale. The healthy children included in our study did not suffer from any diseases which required hospitalization and had not undergone any surgical procedures during the previous year. What is more, they had not been treated pharmacologically just before the studies began, and their results of routine laboratory tests, i.e., blood cell morphology, blood levels of cholesterol, glucose, and creatinine, were normal for this age group (Table 1).

### 2.2. The Assay of the Concentration of PIICP, CTXII, and TNF-α

PIICP, CTXII, and TNF-α levels were measured using blindly tested coded serum samples, in duplicate. The determination of a single parameter was completed within a day; consequently the inter-assay variation was insignificant. Enzymatic immunoassays (ELISA) were used to quantify the markers of cartilage transformations, following the manufacturer’s protocol. Serum concentrations of PIICP were determined with Procollagen II C-Terminal Propeptide (PIICP) ELISA Kit by Cloud-Clone Corp. (Houston, TX, USA), with a minimum detection of 12.5 pg/mL. Similarly, the determination of serum CTXII concentration was performed with the Cross Linked C-Telopeptide of Type II Collagen (CTXII) ELISA Kit by Cloud-Clone Corp. (Huston, TX, USA), with a minimum detection of 48.4 pg/mL. Serum TNF-α concentration was determined using the TNF alpha Human ELISA Kit, manufactured by Thermo Fisher Scientific, Inc. (Waltham, MA, USA), with a minimum detection of 1.7 pg/mL.

These studies were approved by the Local Ethics Committee of the Medical University of Silesia in Katowice (KNW/0022/KB/81/15). Informed consent was obtained from the participants or their legal guardians, according to the ethical guidelines of the Declaration of Helsinki, as part of the continuation of our earlier research [16].

### 2.3. Statistical Analysis

A statistical analysis was carried out using Statistica 13.0 package (StatSoft, Cracow, Poland). The normality of distribution was verified with the Shapiro–Wilk test. The homogeneity of variance was assessed by the Levene’s test. Since the variables were normally distributed, they are presented as the mean and standard deviation (±SD). One-way ANOVA and the post hoc Dunnett tests were used to determine whether the differences between means for patients and controls were significant. In order to compare the same parameters in each patient before treatment and after the therapy period, the paired Student’s t-test was used. What is more, the Student’s t-test was used to evaluate the differences between untied variables. The Pearson’s correlation coefficient, modified by the multi-testing Bonferroni correction, was employed for the statistical analysis of correlations between variables. *p*-values of < 0.05 were considered as significant.

## 3. Results

### 3.1. The Serum Levels of PIICP in Healthy Children and JIA Patients

Based on the obtained results in all individuals with untreated JIA, we found a significant increase of serum concentrations of PIICP. As shown in Table 2, the untreated patients from the A subgroup (those who later demonstrated sensitivity to treatment) had higher levels of these propeptides, compared to the controls by 39% (*p* = 0.0004), while the untreated patients from the B subgroup (those who later did not respond to treatment) by 33% (*p* = 0.0000). PIICP levels in both subgroups of patients before treatment were comparable (*p* = 0.529).

It was observed that the therapy modifying the course of inflammation, which was administered to JIA patients, and contributing to clinical improvement, resulted also in a significant decrease (*p =* 0.0000, by 37%) in serum levels of PIICP in patients with inactive JIA (A’), vs. the pre-treatment situation. In this group of patients, the pharmacological therapy normalized concentrations of the assessed marker. On the other hand, blood concentrations of PIICP in patients not responding to the therapy (B’, i.e., active disease) remained significantly higher, as compared to concentrations in both the controls (*p* = 0.0000, by 67%), and the untreated JIA patients (B) (*p* = 0.0000, by 26%), as well as in the treated remissive JIA (A, i.e., inactive disease) patients (*p* = 0.0000, by 65%) (Table 2).

**Figure 1:**
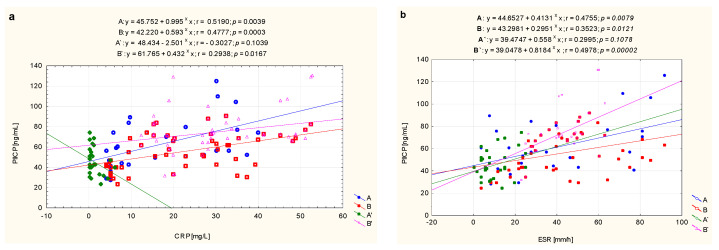
Graphical analysis of the strength of the linear relationship between serum concentrations of PIICP and CRP (**a**) as well as ESR (**b**) in JIA patients, i.e., JIA patients before treatment—later responding to treatment (A), JIA patients before treatment—later not responding to treatment (B), as well as the same patients after treatment obtaining clinical improvement (A’) and without clinical improvement (B’).

The analysis of the relations between PIICP concentration and the inflammatory indicators which were routinely evaluated, i.e., CRP and ESR, revealed a significant relationship with the listed variables in children with newly diagnosed, untreated JIA. The values in the A subgroup were as follows: PIICP and CRP (r = 0.519, *p* = 0.004,), and ESR (r = 0.475, *p* = 0.010), respectively. While in the B subgroup they were: PIICP and CRP (r = 0.478, *p* = 0.000), and ESR (r = 0.352, *p* = 0.012), respectively. What is more, we recorded insignificant correlations between PIICP and CRP (r = −0.303, *p* = 0.104) as well as PIICP and ESR (r = 0.299, *p* = 0.108) in treated patients with JIA whose clinical condition had stabilized (A’, i.e., inactive disease). On the contrary, correlations between PIICP and CRP (r = 0.294, *p* = 0.017) as well as PIICP and ESR (r = 0.498, *p* = 0.000) were observed in the treated patients with active JIA (B’) (Figure 1).

### 3.2. The Serum Levels of CTXII in Healthy Children and JIA Patients

The obtained results also indicated a significant difference in the concentration of the CTXII between the group of patients with JIA who were not subjected to the therapy and the individuals from the control group. The concentration of the evaluated marker in patients with arthropathy, before treatment, was significantly higher both in the A subgroup (*p* = 0.0000, by 76%) as well as in the B subgroup (*p* = 0.0000, by 75%), than in healthy individuals. What is more, it was observed that the therapy administered to JIA patients resulted in the significant decrease (*p* = 0.0009, by 31%) in serum levels of assessed telopeptide only in JIA remissive children (A’, inactive disease), vs. the pre-treatment situation. However, the concentrations of CTXII in the blood of children with clinically stabilized disease (A’) were still statistically (*p* = 0.045, by 21%) different from those in the serum of healthy children. Similarly, the treated patients, who did not show clinical improvement (B’), still had higher serum levels of the mentioned parameter, as compared to the controls (95%, *p* = 0.0000) and treated remissive patients (A’, inactive disease) (61%, *p* = 0.0000). The serum CTXII levels in the treated patients with the active disease (B’) corresponded to concentrations in the untreated patients (B) (*p* = 0.1188) (Table 2).

**Figure 2:**
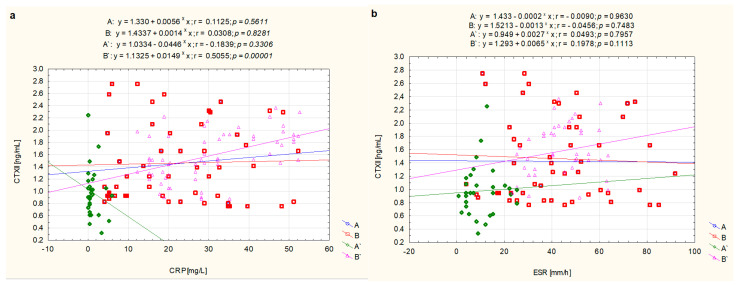
Graphical analysis of the strength of the linear relationship between serum concentrations of CTXII and CRP (**a**) as well as ESR (**b**) in JIA patients, i.e., JIA patients before treatment—later responding to treatment (A), JIA patients before treatment—later not responding to treatment (B), as well as the same patients after treatment obtaining clinical improvement (A’) and without clinical improvement (B’).

The analysis of the relations between CTXII concentration and CRP as well as ESR did not reveal a relationship between these parameters in all patients with newly diagnosed, untreated JIA as well as in the treated patients JIA whose clinical condition had stabilized (A’, inactive disease). The values in the A subgroup were as follows: CTXII and CRP (r = 0.113, *p* = 0.561), and ESR (r = −0.009, *p* = 0.963), respectively. While in the B subgroup they were: CTXII and CRP (r = 0.031, *p* = 0.828), and ESR (r = −0.046, *p* = 0.748), respectively. In the treated patients with inactive disease (A’) the correlations were: CTXII and CRP (r = −0.184, *p* = 0.331), and ESR (r = 0.049, *p* = 0.796), respectively. We recorded significant correlations between CTXII and CRP (r = 0.506, *p* = 0.0001) in patients with the treated JIA whose clinical condition had not stabilized (B’, active disease). No similar relationship was found for ESR (r = 0.198, *p* = 0.111) (Figure 2).

### 3.3. The Serum Levels of TNF-α in Healthy Children and JIA Patients

The results of the TNF-α serum concentration determination indicated a significant growth in the amount of this cytokine in all patients with JIA before treatment, both in the A (*p* = 0.0006, by 37%) and B (*p* = 0.0000, by 41%) subgroups, compared to the healthy individuals. The applied therapy resulted in a decrease (*p* = 0.0003, by 23%) of TNF-α serum level in patients with remission (A’, inactive disease), vs. their pre-treatment status. Concentrations of the assessed cytokine in the blood of children with clinically stabilized disease (A’) were not statistically (*p* = 0.3922) different from those in the serum of healthy children. What is more, the blood concentrations of the evaluated marker in the treated patients showing no clinical improvement (B’, active disease) were statistically higher, as compared to the TNF-α serum level in both the healthy children (*p* = 0.0000, by 79%) and in the patients with JIA who were not subjected to the therapy (B, *p* = 0.0063, by 27%), as well as the treated patients with inactive JIA (A’, *p* = 0.0001, by 70%) (Table 2).

**Figure 3:**
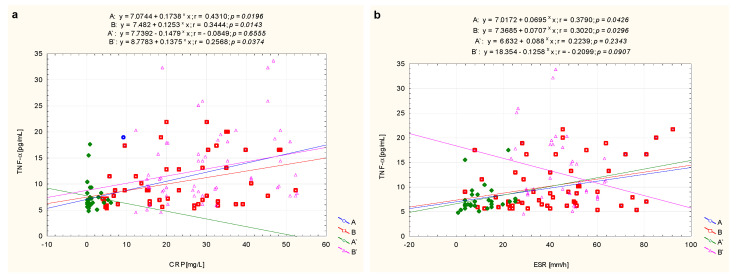
Graphical analysis of the strength of the linear relationship between serum concentrations of TNF-α and CRP (**a**) as well as ESR (**b**) in JIA patients, i.e., JIA patients before treatment—later responding to treatment (A), JIA patients before treatment—later not responding to treatment (B), as well as the same patients after treatment obtaining clinical improvement (A’) and without clinical improvement (B’).

The analysis of the relations between TNF-α concentrations and CRP and ESR revealed a relationship with the listed variables in children with newly diagnosed, untreated JIA. The values in the A subgroup were as follows: TNF-α and CRP (r = 0.431, *p* = 0.020), and ESR (r = 0.379, *p* = 0.043), respectively. While in the B subgroup the values were TNF-α and CRP (r = 0.344, *p* = 0.014), and ESR (r = 0.302, *p* = 0.030), respectively. We recorded insignificant correlations between TNF-α and CRP (r = −0.085, *p* = 0.655) and ESR (r = 0.223, *p* = 0.234) in patients with JIA whose clinical condition had stabilized (A’, i.e., inactive disease). What is more, no relationship was found between TNF-α and ESR (r = −0.210, *p* = 0.091) in treated patients with active JIA. In the latter patients, correlations between TNF-α and CRP (r = 0.257, *p* = 0.037) were found (Figure 3).

### 3.4. The Correlations between Markers of Metabolic Changes of Cartilage and TNF-α

**Figure 4:**
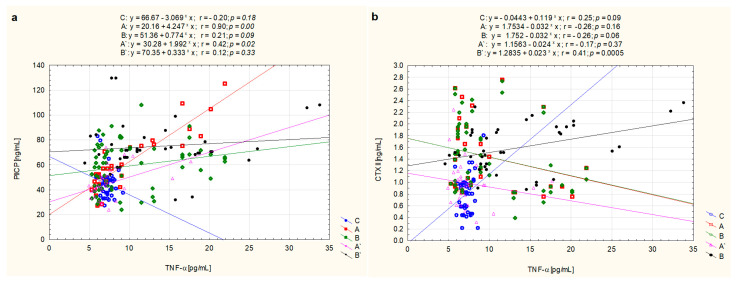
Graphical analysis of the strength of the linear relationship between serum concentrations of TNF-α and PIICP (**a**) as well as CTXII (**b**) in healthy children (C, control subjects) and JIA patients, i.e., JIA patients before treatment—later responding to treatment (A), JIA patients before treatment—later not responding to treatment (B), as well as the same patients after treatment obtaining clinical improvement (A’) and without clinical improvement (B’).

The presented analysis of the strength of the linear relationship between PIICP concentration and the concentration of TNF-α in the blood of untreated JIA children from the A subgroup, described by the Pearson correlation coefficient r = 0.898, (*p* = 0.0001), showed a high correlation between these parameters. However, between the above-mentioned variables in the group of the same patients whose clinical condition stabilized after treatment (A’), the correlation coefficient r = 0.420 (*p* = 0.021) was found. No significant correlations between PIICP and TNF-α blood concentrations in JIA children from the B (r = 0.208, *p* = 0.091) and B’ (r = 0.121, *p* = 0.332) subgroups, were found [(Figure 4a)].

Analyzing the strength of the relation between CTXII and TNF-α, a relationship described by the Pearson correlation coefficient r = 0.410 (*p* = 0.0005) between these variables was demonstrated in patients from the B’ subgroup, i.e., treated patients who did not achieve clinical improvement. What is more, as presented graphically in Figure 4b, in other subgroups of patients, i.e., A (r = −0.256, *p* = 0.163), A’ (r = −0.174, *p* = 0.367), as well as B (r = −0.264, *p* = 0.061), no significant correlations were found.

## 4. Discussion

In the course of JIA, articular cartilage ECM disorders occur [10]. We have shown a significant increase in the serum concentration of both PIICP, which is an indicator of the biosynthesis of cartilage structures, and CTXII, which reflects the degree of resorption of this tissue, in relation to the concentration of the assessed markers in the serum of healthy children. These results indicated a significantly enhanced metabolism of type II collagen, i.e., the dominant organic component of cartilage structures. This transformation involves the processes of synthesis and degradation that do not remain in equilibrium in afflicted children. It should be mentioned that proper, age-specific metabolism of cartilage tissue requires a synchronized and systematic sequence of changes consisting in both the formation and degradation of the mentioned tissue, with the increase of formation during the developmental ages [8,9,17]. Although in this study an increased type II collagen synthesis was found in untreated children with JIA, it seems that at the onset of clinical symptoms of the disease the process of this protein synthesis does not compensate for its degradation. The above thesis cannot be verified by comparing our research results with the results of other authors, because thus far the quantitative assessment of PIICP has not been the subject of research conducted in children with JIA. The assessment of PIICP was performed in adults with other cartilage diseases [18,19,20,21,22,23,24]. Researchers have suggested that an increase in collagen synthesis, shown by PIICP concentration, is related to the type of arthropathy and the severity of the inflammatory process [22,23]. According to the authors, the maximum amount of biosynthesis of the discussed fibrous protein occurs long before the appearance of radiological changes indicating the development of arthropathy. It also occurs during the disease phase characterized by symptoms of increased degradation of CII, aggrecan, and other components of the ECM [24]. The above hypothesis was confirmed by the research of Sugiyama et al. [20]. This degradation takes place particularly rapidly in the surface layers of cartilage, facilitating the reduction of CII content. During this time, the deeper cartilage layers become the source of a “regenerative response”, which is characterized by increased fibrous protein biosynthesis [20,24].

It is known that during the early stages of osteoarthritis, and possibly in the course of JIA, chondrocytes synthesize more CII to compensate for its loss. It has been observed that the newly synthesized molecules are often damaged, which hinders the effective regeneration of the ECM cartilage. As a consequence, damaged collagen fibers may accumulate in a JIA child, which limits the “reparative” proliferation of chondrocytes. Regenerative phenomena are also inhibited by proteoglycans including aggrecan, decorin, or biglycan, accumulated in the deeper layers of ECM [24,25]. On the other hand, proteoglycans are “lost” from the articular surface, which, together with the degradation of CII that is not compensated for by its synthesis, may contribute to the development of disorders of the structure and function of articular cartilage in children. It is believed that aggrecan degradation is a phenomenon that occurs in the early stages of arthritis in children and is likely to precede irreversible changes in the weaving structure of collagen fibers [25,26]. Therefore, the prompt implementation of therapy in patients with JIA, based on the results of diagnostic tests, which also define of severity and/or the progress of CII degradation, may prevent the degradation of joint structures.

In the case of markers of metabolism of the CII, it was shown that the C-terminal telopeptide of the α chain of type II collagen may be a potential indicator of the progress of cartilage degenerative changes. The usefulness of CTXII in the above assessment, however only in adults, is confirmed by the results of studies by numerous authors [12,27,28,29,30,31]. These studies showed a significant increase in the concentration of telopeptide both in the urine and synovial fluid of people with various types of systemic connective tissue diseases, regardless of the causes. This points to the possibility of using the quantitative characterization of CTXII as a single indicator for assessment in combination with other markers of cartilage turnover, e.g., cartilage oligomeric matrix protein (COMP), in assessing both the severity of degenerative changes in the joints and in assessing the response to chondroprotective treatment [12,30,31].

As demonstrated in our study, a significant increase in the concentration of CTXII occurs in the blood of children diagnosed with JIA, especially in those with high disease activity. Struglics et al. [32] also confirmed the increased degradation of CII in the course of JIA, manifested by an increase in the amount of circulating type II collagen epitope (C2C) in the blood of patients. According to the authors, elevated levels of destruction biomarkers in JIA, including C2C or COMP, compared to healthy children indicate potential to serve as clinical tools for destructive joint disease [32].

Disturbances in the metabolism of the CII in the course of JIA, confirmed by the results of this study, seem to be complex and largely conditioned by the direct action of inflammatory mediators, including TNF-α [14,33]. We documented, like Funk et al. [34], significantly higher concentrations of TNF-α in the blood of children with active JIA. Moreover, as demonstrated by us, the relationship of TNF-α with cartilage turnover markers seems to indicate that the evaluated proinflammatory factor is particularly associated with PIICP transformations. It is known that TNF-α stimulates the synthesis of PDGF as well as vascular endothelial growth factors (VEGFs), recognized stimulants of collagen biosynthesis [10,35]. On the other hand, TNF-α is able to eliminate the anabolic effects of other stimulants, such as TGF-β1 or IGF-1 [36,37]. However, it is not clear which effects of TNF-α are dominant in children with JIA. Moreover, in the course of the discussed arthropathy, different trends in the concentrations of the above-mentioned growth factors are observed. In the blood of patients with untreated JIA a significant increase of PDGF-BB, TGF-β1, and a decrease of VEGF levels was stated. Additionally, IGF-1 levels slightly decreased or they were comparable to those observed in healthy children [38,39,40,41]. However, the clinical presentation of patients with newly diagnosed, untreated JIA seems to indicate that at the time of onset of arthropathy the syntheses of the ECM components does not balance the magnitude of their degradation.

The degradation of cartilage in children with JIA is initiated by the influence of proinflammatory cytokines, including IL-1β, IL-6, and especially the above-mentioned TNF-α. The TNF-α enhances the influx of leukocytes into the synovium, stimulating the proliferation and differentiation of both T and B lymphocytes, and also changing the mutual interactions between osteoclasts, osteoblasts, and cells of the immune system, inducing the synthesis of proteinases by chondrocytes and leading to the destruction of ECM components [33,34].

Matrix metalloproteinases are among the proteolytic factors involved in the depolymerization of the collagen network of articular cartilage [42,43,44,45,46], whose activity is reflected in the high concentrations of CTXII observed in the blood of patients with active JIA. It is assumed that MMP-1, -8, -13, -14, and cathepsin K are the enzymes responsible for the first stage of collagen degradation, while MMP-2 and -9 additionally digest degraded collagen fibers. Moreover, MT1-MMP (1-membrane MMP type), MT3-MMP, and possibly MT2-MMP constitute another group of factors whose interaction contributes to the loss of collagen [25,26,42,43,44,45,46]. The thesis suggesting the increased enzymatic degradation of ECM fibrous proteins is confirmed by significantly higher concentrations of MMP-1, -2, -3, or -9 demonstrated in the blood, synovial fluid, and saliva of children with JIA, which are not balanced by the concentrations of their tissue inhibitors, i.e., TIMP-1 and -2 [10,25,46,47,48]. It is worth adding that the high concentrations of MMP-2 and -9 in the synovial fluid and in the serum of children with JIA prove the presence of the increased degradation of type II collagen already in the early stages of arthropathy [26,49].

Moreover, ROSs are also involved in the extracellular degradation of ECM components [10,50]. ROSs, secreted by cells of the immune system which infiltrate the synovium, lead to the degradation of the cartilage ECM, both directly through the depolymerization of collagen fibers and indirectly through the activation of MMPs [10,50,51]. The aforementioned activation of proteinases occurs through the ROS-dependent stimulation of the signaling network, co-created by numerous molecules, including p38, ERK, JNKs, p65, Akt, or Nrf2. Moreover, the stimulation of signaling molecules, sensitive to ROSs, also reduces the expression of genes encoding protein components of ECM and increases the expression of those genes that code for pro-inflammatory compounds, including TNF-α or cyclooxygenase-2 (COX -2) [51].

The observed changes in the levels of cartilage turnover markers in the blood of children with JIA are also associated with the autoimmune process. The presence of autoantibodies against ECM components in the blood of sick children, including antibodies to type II collagen (anti-CII p/c), seem to confirm the latter hypothesis [52,53]. It is believed that the presence of anti-CII antibodies in the blood of patients at an early stage of disease may predict later joint damage [53].

As can be concluded from the above data, the pathways leading to the increase in PIICP and CTXII observed in untreated children with JIA are complex and often interdependent. However, the demonstrated profile of the assessed compounds in the blood of children with clinically controlled JIA clearly indicates that the inflammatory process is the link between all the mechanisms of pathogenetic changes leading to the development of arthropathy. It has been shown that the use of drugs modifying the course of the disease, which leads in some patients to quenching of the inflammatory process, simultaneously contribute to lowering the concentrations of both PIICP and CTXII in the blood. On the other hand, the concentrations of the assessed markers in the blood of patients unresponsive to the applied therapy were significantly different from those of patients with clinically controlled JIA. The profile of the concentration of markers of ECM remodeling observed in the treated children with JIA, whose condition was not improved, suggests the need to continue a therapy aimed at protecting the patient against possible disability. As a result of disorders of ECM transformations, disorders of growth and maturation may appear in children with JIA. Moreover, longstanding temporomandibular arthritis, affecting 39–75% of patients with JIA, can result in pain, limitation in mouth opening, facial asymmetry, retrognathism, and malocclusion [54,55]. Early diagnosis leading to treatment may minimize or prevent these secondary effects [54].

## 5. Conclusions

In summary, the significant changes observed in serum PIICP during JIA therapy suggest its potential diagnostic utility in the monitoring of disease activity and that it may be used to assess treatment towards remission. The reference values for PIICP concentrations must be established, appropriate for individual age groups of children with JIA, in order for its use in monitoring the condition of cartilage in JIA patients. Understanding the changes in CII metabolism over the course of the discussed arthritis may allow the implementation of both new diagnostic tools and new therapeutic strategies in children with JIA.

Moreover, our results confirm the suggestion that the use of TNF-α inhibitors in JIA therapy does not guarantee remission in all patients qualified for biological treatment. It is necessary to jointly assess the concentration of TNF-α with IL-1β and IL-6, which could allow the identification of a group of patients whose severe course or frequent exacerbations of the disease are associated with increased levels of IL-1β and/or IL-6, in the presence of low TNF-α concentrations.

## Figures and Tables

**Table 1:** The clinical characteristics of control subjects and JIA patients.

Parameter	Control Subjects (*n* = 45)	JIA Patients before Treatment (*n* = 96)	JIA Patients after Treatment	
			Inactive Disease (*n* = 30)	Active Disease (*n* = 66)
Age (years)	8.25 ± 2.03	8.23 ± 3.48	8.71 ± 3.70	7.95 ± 2.51
Sex, female/male	34/11	77/19	19/11	58/8
JADAS-27	-	18 ± 8.66	4 ± 2.48 ^b^	15 ± 4.57
BMI (kg/m^2^)	18.34 ± 2.12	16.18 ± 2.14 ^a^	18.02 ± 3.55 ^b^	16.67 ± 3.12 ^a^
WBC (10^3^/µL)	7.85 ± 2.34	14.66 ± 4.59 ^a^	6.45 ± 2.70	9.72 ± 2.18 ^b^
RBC (10^6^/µL)	4.75 ± 0.32	4.26 ± 0.42	4.62 ± 0.36	4.02 ± 0.33 ^a^
Hb (g/dL)	14.08 ± 0.74	11.25 ± 1.78 ^a^	12.94 ± 1.53 ^a,b^	12,02 ± 1.27 ^a,b^
Ht (%)	40.68 ± 3.26	35.67 ± 3.52 ^a^	37.22 ± 7.51 ^a,b^	37.19 ± 3.57 ^a,b^
PLT (10^3^/µL)	284.42 ± 68.22	398.26 ± 111.87 ^a^	359.26 ± 80.06 ^b^	344.32 ± 70.15
Total cholesterol (mM/L)	4.32 ± 0.84	4.69 ± 1.39 ^a^	4.27 ± 1.55 ^b^	4.48 ± 0.69
Glucose (mM/L)	4.21 ± 0.38	4.18 ± 1.26	4.44 ± 0.56 ^b^	4.51 ± 0.93 ^b^
Creatinine (µM/L)	61.42 ± 12.45	77.58 ± 9.21 ^a^	64.35 ± 14.57 ^a,b^	82.58 ± 1.11 ^a,b^
CRP (mg/L)	1.20 ± 1.39	19.66 ± 21.68 ^a^	3.57 ± 0.62 ^b^	12.47 ± 16.88 ^a,c^
ESR (mm/h)	9.22 ± 7.41	41.66 ± 22.04 ^a^	12.01 ± 5.15 ^b^	24.95 ± 15.89 ^a,c^
ANA	-	57% (positive)	57% (positive)	57% (positive)
RF	-	100% (negative)	100%(negative)	100% (negative)

Results are expressed as mean ± SD; ^a^ *p* < 0.05 compared to control group; ^b^ *p* < 0.05 compared to untreated JIA patients; ^c^ *p* < 0.05 compared to treated JIA patients (inactive disease); BMI, body mass index; WBC, white blood cell; RBC, red blood cell; Hb, hemoglobin; Ht, hematocrit; PLT, platelet; CRP, C-reactive protein; ESR, erythrocyte sedimentation rate; ANA, antinuclear antibodies; RF, rheumatoid factor.

**Table 2:** The distribution patterns of serum PIICP, CTXII, and TNF-α in healthy individuals (control subjects) and juvenile idiopathic arthritis (JIA) patients, i.e., JIA patients before treatment—later responding to treatment (A), JIA patients before treatment—later not responding to treatment (B), as well as the same patients after treatment obtaining clinical improvement (A’) and without clinical improvement (B’).

Parameter	Control Subjects (*n* = 45)	JIA Patients before Treatment		JIA Patients after Treatment	
		A (*n* = 30)	B (*n* = 66)	A’ (Inactive Disease) (*n* = 30)	B’ (Active Disease) (*n* = 66)
PIICP (ng/mL)	44.62 ± 10.66	62.00 ± 23.48 ^a^	59.18 ± 18.71 ^b^	45.33 ± 13.10 ^e^	74.64 ± 18.81 ^a,f,i^
CTX II (ng/mL)	0.81 ± 0.21	1.43 ± 0.75 ^c^	1.42 ± 0.59 ^c^	0.98 ± 0.32 ^d,g^	1.58 ± 0.67 ^c,i^
TNF-α (pg/mL)	7.19 ± 0.71	9.85 ± 4.96 ^d^	10.11 ± 5.02 ^d^	7.56 ± 2.76 ^e^	12.88 ± 6.86 ^c,h,j^

Results are expressed as mean ± SD; PIICP, procollagen II C-terminal propeptide; CTX II, C-telopeptide of type II collagen; TNF-α, tumor necrosis factor α; (^a^ *p* < 0.0005, ^b^ *p* < 0.00001, ^c^ *p* < 0.000001, ^d^ *p* < 0.001 compared to control group; ^e^ *p* < 0.0005, ^f^ *p* < 0.00001, ^g^ *p* <0.001, ^h^ *p* < 0.01 compared to untreated JIA patients; ^i^ *p* < 0.0000005, ^j^ *p* < 0.0001 compared to treated JIA patients (inactive disease).

## Data Availability

The datasets analyzed or generated during the study are available from the authors.
